# Supplementary material for: Modeling glioblastoma heterogeneity as a dynamic network of cell states
Source: Mol Syst Biol. 2021 Sep 16;17(9):e10105. doi: 10.15252/msb.202010105 (PMC8444284; doi:10.15252/msb.202010105)
Supplement: Supplementary file 6 — Source Data for Figure 5 [file MSB-17-e10105-s004.zip › Figure5A_sourcedata/GSEA_3017/nosigdb_stateC.GseaPreranked.1622795002785/DOROSZKO_U3054MG_CLUSTER_4.html]

Details for gene set DOROSZKO\_U3054MG\_CLUSTER\_4[GSEA]

|  || Dataset | state13017 |
| Phenotype | NoPhenotypeAvailable |
| Upregulated in class | na\_pos |
| GeneSet | DOROSZKO\_U3054MG\_CLUSTER\_4 |
| Enrichment Score (ES) | 0.78095853 |
| Normalized Enrichment Score (NES) | 6.651354 |
| Nominal p-value | 0.0 |
| FDR q-value | 0.0 |
| FWER p-Value | 0.0 |
Table: GSEA Results Summary

  

Fig 1: Enrichment plot: DOROSZKO\_U3054MG\_CLUSTER\_4      
 Profile of the Running ES Score & Positions of GeneSet Members on the Rank Ordered List

  

| PROBE | GENE SYMBOL | GENE\_TITLE | RANK IN GENE LIST | RANK METRIC SCORE | RUNNING ES | CORE ENRICHMENT || 1 | SYNE2 |  |  | 0 | 0.839 | 0.0201 | Yes |
| 2 | HES6 |  |  | 5 | 0.694 | 0.0286 | Yes |
| 3 | ATAD2 |  |  | 7 | 0.649 | 0.0421 | Yes |
| 4 | FAM111B |  |  | 8 | 0.616 | 0.0569 | Yes |
| 5 | HIST1H4C |  |  | 9 | 0.603 | 0.0714 | Yes |
| 6 | CENPU |  |  | 10 | 0.588 | 0.0855 | Yes |
| 7 | HIST1H1D |  |  | 12 | 0.583 | 0.0974 | Yes |
| 8 | FAM111A |  |  | 13 | 0.576 | 0.1112 | Yes |
| 9 | EZH2 |  |  | 14 | 0.574 | 0.1250 | Yes |
| 10 | CCNE2 |  |  | 15 | 0.570 | 0.1387 | Yes |
| 11 | CLSPN |  |  | 17 | 0.561 | 0.1501 | Yes |
| 12 | C21orf58 |  |  | 18 | 0.559 | 0.1635 | Yes |
| 13 | RRM2 |  |  | 19 | 0.542 | 0.1765 | Yes |
| 14 | HMGB2 |  |  | 20 | 0.534 | 0.1893 | Yes |
| 15 | MCUB |  |  | 21 | 0.533 | 0.2021 | Yes |
| 16 | XRCC2 |  |  | 26 | 0.495 | 0.2058 | Yes |
| 17 | CHAF1A |  |  | 27 | 0.494 | 0.2177 | Yes |
| 18 | PCLAF |  |  | 29 | 0.484 | 0.2272 | Yes |
| 19 | UBE2T |  |  | 31 | 0.475 | 0.2366 | Yes |
| 20 | HIST1H1B |  |  | 35 | 0.460 | 0.2415 | Yes |
| 21 | SAMD1 |  |  | 36 | 0.457 | 0.2525 | Yes |
| 22 | CDCA7L |  |  | 37 | 0.453 | 0.2633 | Yes |
| 23 | GMNN |  |  | 38 | 0.453 | 0.2742 | Yes |
| 24 | ORC6 |  |  | 39 | 0.450 | 0.2850 | Yes |
| 25 | MMS22L |  |  | 40 | 0.448 | 0.2958 | Yes |
| 26 | ESCO2 |  |  | 43 | 0.440 | 0.3022 | Yes |
| 27 | UHRF1 |  |  | 45 | 0.436 | 0.3106 | Yes |
| 28 | RAD51AP1 |  |  | 46 | 0.435 | 0.3211 | Yes |
| 29 | PCNA |  |  | 48 | 0.435 | 0.3295 | Yes |
| 30 | TMPO |  |  | 49 | 0.432 | 0.3399 | Yes |
| 31 | DTL |  |  | 50 | 0.430 | 0.3502 | Yes |
| 32 | HIST1H1E |  |  | 51 | 0.430 | 0.3605 | Yes |
| 33 | TOP2A |  |  | 52 | 0.426 | 0.3707 | Yes |
| 34 | SPC25 |  |  | 53 | 0.415 | 0.3807 | Yes |
| 35 | BRCA1 |  |  | 54 | 0.410 | 0.3905 | Yes |
| 36 | DHFR |  |  | 55 | 0.405 | 0.4002 | Yes |
| 37 | WDR76 |  |  | 56 | 0.403 | 0.4099 | Yes |
| 38 | TYMS |  |  | 60 | 0.390 | 0.4131 | Yes |
| 39 | PHIP |  |  | 61 | 0.387 | 0.4224 | Yes |
| 40 | RRM1 |  |  | 62 | 0.386 | 0.4317 | Yes |
| 41 | SMC2 |  |  | 63 | 0.386 | 0.4409 | Yes |
| 42 | E2F1 |  |  | 67 | 0.380 | 0.4439 | Yes |
| 43 | E2F7 |  |  | 69 | 0.377 | 0.4509 | Yes |
| 44 | PSIP1 |  |  | 73 | 0.374 | 0.4538 | Yes |
| 45 | NASP |  |  | 75 | 0.371 | 0.4606 | Yes |
| 46 | POLD3 |  |  | 76 | 0.367 | 0.4694 | Yes |
| 47 | CENPH |  |  | 77 | 0.366 | 0.4782 | Yes |
| 48 | SMC4 |  |  | 78 | 0.365 | 0.4870 | Yes |
| 49 | ITGB3BP |  |  | 79 | 0.364 | 0.4957 | Yes |
| 50 | CDCA5 |  |  | 81 | 0.361 | 0.5023 | Yes |
| 51 | GINS2 |  |  | 83 | 0.360 | 0.5089 | Yes |
| 52 | KLHL23 |  |  | 84 | 0.359 | 0.5175 | Yes |
| 53 | BARD1 |  |  | 86 | 0.357 | 0.5241 | Yes |
| 54 | MIS18BP1 |  |  | 89 | 0.355 | 0.5285 | Yes |
| 55 | CARHSP1 |  |  | 90 | 0.353 | 0.5370 | Yes |
| 56 | MCM7 |  |  | 91 | 0.352 | 0.5454 | Yes |
| 57 | USP1 |  |  | 92 | 0.352 | 0.5539 | Yes |
| 58 | ASRGL1 |  |  | 93 | 0.350 | 0.5623 | Yes |
| 59 | ATAD5 |  |  | 97 | 0.345 | 0.5644 | Yes |
| 60 | FEN1 |  |  | 98 | 0.342 | 0.5726 | Yes |
| 61 | NUSAP1 |  |  | 100 | 0.340 | 0.5787 | Yes |
| 62 | NCAPD3 |  |  | 103 | 0.334 | 0.5826 | Yes |
| 63 | MCM8 |  |  | 104 | 0.331 | 0.5906 | Yes |
| 64 | SLBP |  |  | 106 | 0.331 | 0.5965 | Yes |
| 65 | GINS4 |  |  | 112 | 0.325 | 0.5941 | Yes |
| 66 | MCM3 |  |  | 114 | 0.324 | 0.5998 | Yes |
| 67 | H2AFX |  |  | 115 | 0.322 | 0.6075 | Yes |
| 68 | HIRIP3 |  |  | 116 | 0.321 | 0.6152 | Yes |
| 69 | PSMC3IP |  |  | 118 | 0.319 | 0.6208 | Yes |
| 70 | TIMELESS |  |  | 119 | 0.319 | 0.6285 | Yes |
| 71 | CCDC34 |  |  | 120 | 0.318 | 0.6361 | Yes |
| 72 | HAT1 |  |  | 121 | 0.318 | 0.6438 | Yes |
| 73 | SMC1A |  |  | 125 | 0.312 | 0.6451 | Yes |
| 74 | DNMT1 |  |  | 127 | 0.311 | 0.6505 | Yes |
| 75 | FBXO5 |  |  | 130 | 0.308 | 0.6538 | Yes |
| 76 | KIF15 |  |  | 136 | 0.302 | 0.6508 | Yes |
| 77 | NCAPG |  |  | 137 | 0.301 | 0.6581 | Yes |
| 78 | RFC4 |  |  | 138 | 0.300 | 0.6653 | Yes |
| 79 | CENPK |  |  | 139 | 0.299 | 0.6724 | Yes |
| 80 | LMNB1 |  |  | 142 | 0.293 | 0.6754 | Yes |
| 81 | ASF1B |  |  | 143 | 0.291 | 0.6824 | Yes |
| 82 | HPF1 |  |  | 147 | 0.289 | 0.6831 | Yes |
| 83 | SUPT16H |  |  | 153 | 0.286 | 0.6798 | Yes |
| 84 | HDGFL3 |  |  | 154 | 0.285 | 0.6866 | Yes |
| 85 | RFC3 |  |  | 156 | 0.283 | 0.6914 | Yes |
| 86 | CEP78 |  |  | 159 | 0.281 | 0.6940 | Yes |
| 87 | ZNF93 |  |  | 160 | 0.281 | 0.7008 | Yes |
| 88 | MYBL2 |  |  | 164 | 0.278 | 0.7013 | Yes |
| 89 | H2AFY |  |  | 165 | 0.278 | 0.7080 | Yes |
| 90 | VRK1 |  |  | 169 | 0.275 | 0.7084 | Yes |
| 91 | CIP2A |  |  | 170 | 0.272 | 0.7150 | Yes |
| 92 | HELLS |  |  | 171 | 0.271 | 0.7215 | Yes |
| 93 | C19orf48 |  |  | 173 | 0.271 | 0.7259 | Yes |
| 94 | MAD2L1 |  |  | 174 | 0.270 | 0.7324 | Yes |
| 95 | RBBP8 |  |  | 177 | 0.267 | 0.7347 | Yes |
| 96 | POLA1 |  |  | 179 | 0.266 | 0.7390 | Yes |
| 97 | MSH2 |  |  | 180 | 0.265 | 0.7454 | Yes |
| 98 | DDX11 |  |  | 181 | 0.265 | 0.7517 | Yes |
| 99 | RMI1 |  |  | 183 | 0.263 | 0.7560 | Yes |
| 100 | NAP1L4 |  |  | 184 | 0.262 | 0.7623 | Yes |
| 101 | FANCL |  |  | 185 | 0.261 | 0.7685 | Yes |
| 102 | NCAPG2 |  |  | 187 | 0.260 | 0.7727 | Yes |
| 103 | MCM4 |  |  | 189 | 0.259 | 0.7769 | Yes |
| 104 | CDK1 |  |  | 191 | 0.255 | 0.7810 | Yes |
| 105 | PMP2 |  |  | 424 | -0.337 | 0.3146 | No |
| 106 | MIF |  |  | 442 | -0.347 | 0.2882 | No |
| 107 | PDLIM4 |  |  | 466 | -0.370 | 0.2500 | No |
| 108 | MT2A |  |  | 566 | -0.575 | 0.0613 | No |
Table: GSEA details [plain text format]

  

Fig 2: DOROSZKO\_U3054MG\_CLUSTER\_4: Random ES distribution      
 Gene set null distribution of ES for **DOROSZKO\_U3054MG\_CLUSTER\_4**
